# Robust inference for the Two-Sample 2SLS estimator^☆^

**DOI:** 10.1016/j.econlet.2016.06.033

**Published:** 2016-09

**Authors:** David Pacini, Frank Windmeijer

**Affiliations:** a Department of Economics, University of Bristol, UK; b IEU, Bristol, UK; c Cemmap, London, UK

**Keywords:** Linear model, Data combination, Instrumental variables, Robust inference, Nonlinear GMM

## Abstract

The Two-Sample Two-Stage Least Squares (TS2SLS) data combination estimator is a popular estimator for the parameters in linear models when not all variables are observed jointly in one single data set. Although the limiting normal distribution has been established, the asymptotic variance formula has only been stated explicitly in the literature for the case of conditional homoskedasticity. By using the fact that the TS2SLS estimator is a function of reduced form and first-stage OLS estimators, we derive the variance of the limiting normal distribution under conditional heteroskedasticity. A robust variance estimator is obtained, which generalises to cases with more general patterns of variable (non-)availability. Stata code and some Monte Carlo results are provided in an Appendix. Stata code for a nonlinear GMM estimator that is identical to the TS2SLS estimator in just identified models and asymptotically equivalent to the TS2SLS estimator in overidentified models is also provided there.

## Highlights

• We derive the variance of the TS2SLS estimator under heteroscedasticity.
• We propose a new robust variance estimator.
• We provide Stata code for the TS2SLS estimator and its robust variance estimator.
• We provide Stata code for an asymptotically equivalent nonlinear GMM estimator.

## 1 Introduction

The Two-Sample Two-Stage Least Squares (TS2SLS) estimator was introduced by Klevmarken (1982) and applies in cases where one wants to estimate the effects of possibly endogenous explanatory variables x on outcome y, but where y and x are not observed in the same data set. Instead, one has observations on outcomes y and instruments z in one sample (sample 1) and on x and z in another (sample 2). Related Two-Sample IV (TSIV) estimators were proposed by Arellano and Meghir (1992) and Angrist and Krueger (1992). Furthermore, Angrist and Krueger (1995) proposed the TS2SLS estimator as a Split-Sample IV (SSIV) estimator. Inoue and Solon (2010) show that the TS2SLS estimator is more efficient than the TSIV estimator of Angrist and Krueger (1992). For further details, see Angrist and Pischke (2009) and the review of Ridder and Moffitt (2007).

This type of data combination estimation method is popular in economics. It is for example used in research on intergenerational mobility, as earnings of different generations are often not observed in the same data set, see the extensive list of references in Jerrim et al. (2014). A further recent application is van den Berg et al. (in press), who investigate the effect of early-life hunger on late-life health and use the two-sample IV approach to deal with imperfect recollection of conditions early in life. Pierce and Burgess (2013) propose the use of the TS2SLS estimator in epidemiology, in particular when estimating the causal relationship between an exposure and an outcome using genetic factors as instrumental variables, so-called Mendelian randomisation, and where obtaining complete exposure data may be difficult due to high measurement costs.

Under certain assumptions, as stated below, the TS2SLS estimator is consistent and has a limiting normal distribution, see e.g.  Klevmarken (1982) and Inoue and Solon (2010). Here we derive the limiting distribution of the TS2SLS estimator under general, unspecified, forms of conditional heteroskedasticity. As the TS2SLS estimator is a simple function of the reduced form parameters for y in sample 1, and the first-stage parameters for x in sample 2, its asymptotic variance is a function of the variances and covariances of these OLS estimators.

The variance of the limiting normal distribution of the TS2SLS estimator is given in (10) below and the formula for a robust estimator of the asymptotic variance is presented in (12). Neither of these have been derived and/or proposed in the literature before. The result in Inoue and Solon (2010) for the conditionally homoskedastic case is similar to our result for that case. They derive the limiting variance of the TS2SLS estimator from the optimal nonlinear GMM estimator. For overidentified models, these two estimators are not the same, but they have the same limiting distribution. Inoue and Solon (2010) did not derive the limiting robust variance for this GMM estimator, but did derive the limiting variance of the efficient two-step GMM estimator under general forms of conditional heteroskedasticity in Inoue and Solon (2005), which is also the approach presented in Arellano and Meghir (1992). Our derivation is different as we focus solely on the TS2SLS estimator as defined below in (5). For the conditional homoskedastic case, our variance estimator differs from the one proposed by Inoue and Solon (2010), as it uses the information from the two samples differently.

Applied researchers have constructed robust standard errors for the just-identified single endogenous regressor case by means of the delta method, see e.g.  Dee and Evans (2003). Our result can be seen as a generalisation of this method to situations with multiple regressors and overidentification. Although we consider here a simple cross-sectional setup, other sampling designs can be accommodated and the result is straightforwardly extended to compute, for example, cluster-robust standard errors.

Our result also generalises to situations outside the standard TS2SLS setup. For example, it can accommodate a model with three explanatory variables where one endogenous variable is observed with the outcome variable in sample 1, but not in sample 2, one explanatory variable is only observed in sample 2 and one endogenous variable is observed in both samples 1 and 2. This is discussed in Section  5 below and we present Stata code for this example and for the standard TS2SLS setup in the Appendix (see Appendix A).

In the next section we present the model, assumptions and the TS2SLS estimator. In Section  3, we present our main results. Section  4 compares our results to those derived for nonlinear GMM. The Appendix also presents Stata code for the GMM estimator.

## 2 Model, assumptions and TS2SLS estimator

The structural linear model of interest is given by

(1) $$y_{i}=x_{i}^{\prime }\beta +\varepsilon_{i}\text{,}$$

but we cannot estimate this model as $y_{i}$ and $x_{i}$ are not jointly observed. Instead, we have two independent samples. In sample 1 we have observations on y and $k_{z}$ exogenous instruments z. Sample 2 contains observations on the $k_{x}$ explanatory variables x and z. Denoting by subscripts 1 and 2 whether the variables are observed in sample 1 or sample 2, in the first sample we observe $\left\{y_{1i},z_{1i}^{\prime }\right\}$ for $i=1,\ldots ,n_{1}$, and in the second sample we observe $\left\{x_{2j}^{\prime },z_{2j}^{\prime }\right\}$ for $j=1,\ldots ,n_{2}$. Throughout we assume that $k_{z}\geq k_{x}$. Other explanatory variables that enter model (1), but that are observed in both samples and are exogenous, including the constant, have been partialled out.

The TS2SLS estimator is derived as follows. From the information in sample 1, we can estimate the reduced form model for $y_{1i}$, given by

(2) $$y_{1i}=z_{1i}^{\prime }\pi_{y1}+u_{1i}\text{.}$$

From sample 2, we can estimate the linear projections

(3) $$x_{2j}=\Pi_{x2}^{\prime }z_{2j}+v_{2j}\text{,}$$

with $\Pi_{x2}=E{\left(z_{2j}z_{2j}^{\prime }\right)}^{-1}E\left(z_{2j}x_{2j}^{\prime }\right)$, a $k_{z}\times k_{x}$ matrix of rank $k_{x}$ by assumption. As (3) is a linear projection, it follows that $E\left(z_{2j}v_{2j}^{\prime }\right)=0$. Although the $x_{1i}$ are not observed, the data generating process for $y_{1i}$ is given by the structural model (1) and hence it and its reduced form are given by

(4) $$y_{1i}=x_{1i}^{\prime }\beta +\varepsilon_{1i}=\left(z_{1i}^{\prime }\Pi_{x1}+v_{1i}^{\prime }\right)\beta +\varepsilon_{1i}=z_{1i}^{\prime }\Pi_{x1}\beta +\varepsilon_{1i}+v_{1i}^{\prime }\beta \text{,}$$

with the linear projection parameters $\Pi_{x1}=E{\left(z_{1i}z_{1i}^{\prime }\right)}^{-1}E\left(z_{1i}x_{1i}^{\prime }\right)$. Again, $E\left(z_{1i}v_{i1}^{\prime }\right)=0$. From (2), (4) it follows that $\pi_{y1}=\Pi_{x1}\beta$ and $u_{1i}=\varepsilon_{1i}+v_{1i}^{\prime }\beta$. Clearly, knowledge of $\pi_{y1}$ and $\Pi_{x1}$ identifies the structural parameters β, and the standard 2SLS estimator in a sample with $y_{1i}$, $x_{1i}$ and $z_{1i}$ all observed combines the information contained in the OLS estimators for $\pi_{y1}$ and $\Pi_{x1}$, denoted by π^y1 and Π^x1 as follows

β^2sls=(Π^x1′Z1′Z1Π^x1)−1Π^x1′Z1′Z1π^y1,

with $Z_{1}$ the $n_{1}\times k_{z}$ matrix $\left[z_{1i}^{\prime }\right]$.

As $x_{1i}$ is not observed, we cannot estimate $\Pi_{x1}$, but we can estimate $\Pi_{x2}$ using the second sample. Denoting the OLS estimator for $\Pi_{x2}$ by Π^x2, the Two-Sample 2SLS estimator is given by

(5) β^ts2sls=(X^1′X^1)−1X^1′y1=(Π^x2′Z1′Z1Π^x2)−1Π^x2′Z1′y1=(Π^x2′Z1′Z1Π^x2)−1Π^x2′Z1′Z1π^y1.

We make the following assumptions:

A1: ${\left\{y_{1i},z_{1i}^{\prime }\right\}}_{i=1}^{n_{1}}$ and ${\left\{x_{2j}^{\prime },z_{2j}^{\prime }\right\}}_{j=1}^{n_{2}}$ are i.i.d. random samples from the same population with finite fourth moments and are independent.
A2: $E\left(z_{1i}z_{1i}^{\prime }\right)=Q_{zz1}$; $E\left(z_{2j}z_{2j}^{\prime }\right)=Q_{zz2}$. $Q_{zz1}$ and $Q_{zz2}$ are nonsingular.
A3: $E\left(z_{1i}x_{1i}^{\prime }\right)$ and $E\left(z_{2i}x_{2i}^{\prime }\right)$ both have rank $k_{x}$.
A4: $E\left(z_{1i}\varepsilon_{1i}\right)=0$.
A5: $E\left(u_{1i}^{2}z_{1i}z_{i1}^{\prime }\right)=\Omega_{y1}$, a finite and positive definite matrix.
A6: $E\left[\left(I_{k_{x}}⊗z_{2j}\right)v_{2j}v_{2j}^{\prime }\left(I_{k_{x}}⊗z_{2j}^{\prime }\right)\right]=E\left(v_{2j}v_{2j}^{\prime }⊗z_{2j}z_{2j}^{\prime }\right)=\Omega_{x2}$, a finite and positive definite matrix. $I_{k_{x}}$ is the identity matrix of order $k_{x}$.
A7: $\lim_{n_{1}\to \infty ,n_{2}\to \infty }\frac{n_{1}}{n_{2}}=\alpha$ for some α>0.

Assumptions A1–A3 and A7 are standard data combination assumptions, see e.g.  Inoue and Solon (2010). Assumptions A2 and A3, combined with A1, result in $E\left(z_{1i}z_{1i}^{\prime }\right)=E\left(z_{2j}z_{2j}^{\prime }\right)$ and $E\left(z_{1i}x_{1i}^{\prime }\right)=E\left(z_{2i}x_{2i}^{\prime }\right)$, and hence $\Pi_{x1}=\Pi_{x2}$. A1–A3 are clearly sufficient, but not necessary conditions for $\Pi_{x1}$ to be equal to $\Pi_{x2}$. The condition $\Pi_{x1}=\Pi_{x2}$ itself is sufficient for consistency of β^ts2sls, and necessary for the limiting normal distribution of n1(β^ts2sls−β) to have a mean of zero. In the derivations below we do not (need to) impose $Q_{zz1}=Q_{zz2}$. The resulting estimator of the variance of β^ts2sls is a simple function of the variances of π^y1 and vec(Π^x2), and this function is unambiguous about which information from which sample is being utilised.

Assumptions A5 and A6 explicitly allow for general forms of heteroskedasticity. The robust variance estimator for β^ts2sls is obtained incorporating robust variance estimators for π^y1 and vec(Π^x2). This was done by Dee and Evans (2003) using the delta method for the just identified single regressor case, i.e.  $k_{x}=k_{z}=1$. The result derived below can be seen as a generalisation of this to multiple regressors and overidentified settings.

## 3 Limiting distribution and variance estimator

The OLS estimators for $\pi_{y1}$ and $\Pi_{x2}$ are given by

π^y1=(Z1′Z1)−1Z1′y1

Π^x2=(Z2′Z2)−1Z2′X2,

with $Z_{1}$ the $n_{1}\times k_{z}$ matrix $\left[z_{1i}^{\prime }\right]$; $Z_{2}$ the $n_{2}\times k_{z}$ matrix $\left[z_{2j}^{\prime }\right]$; $y_{1}$ the $n_{1}$ vector $\left(y_{1i}\right)$ and $X_{2}$ the $n_{2}\times k_{x}$ matrix $\left[x_{2j}^{\prime }\right]$. Under Assumptions A1–A4 and A7 we obtain

plim(π^y1)=E(z1iz1i′)−1E(z1ix1i′)β=πy1=Πx1β=Πx2β;

plim(Π^x2)=E(z2jz2j′)−1E(z2jx2j′)=Πx2,

and hence the TS2SLS estimator is consistent as

(6) plim(β^ts2sls)=plim(1n1Π^x2′Z1′Z1Π^x2)−11n1Π^x2′Z1′Z1π^y1=(Πx2′Qzz1Πx2)−1Πx2′Qzz1πy1=β.

Note that the probability limits obtained here and the limiting distributions derived below are for both $n_{1}\to \infty$ and $n_{2}\to \infty$.

For the derivation of the asymptotic distribution of β^ts2sls, denote $\pi_{x2}=\mathrm{vec}\left(\Pi_{x2}\right)$; π^x2=vec(Π^x2); $\theta ={\left(\begin{array}{cc} \pi_{y1}^{\prime } & \pi_{x2}^{\prime } \end{array}\right)}^{\prime }$and θ^=(π^y1′π^x2′)′. Under Assumptions A1–A7

(7) n1(π^y1−πy1)⟶dN(0,Vπy1);

(8) n2(π^x2−πx2)⟶dN(0,Vπx2),

where

$$V_{\pi_{y1}}=Q_{zz1}^{-1}\Omega_{y1}Q_{zz1}^{-1}\text{;}$$

$$V_{\pi_{x2}}=\left(I_{k_{x}}⊗Q_{zz2}^{-1}\right)\Omega_{x2}\left(I_{k_{x}}⊗Q_{zz2}^{-1}\right)\text{.}$$

Hence

(9) n1(θ^−θ)⟶dN(0,Vθ),

with

$$V_{\theta }=\left[\begin{array}{cc} V_{\pi_{y1}} & 0\\ 0 & \alpha V_{\pi_{x2}} \end{array}\right]\text{.}$$

From the limiting distribution of θ^, the limiting distribution of β^ts2sls is readily obtained and we give a simple proof in the Appendix (see Appendix A). Our main result is:

Under Assumptions A1–A7, the limiting distribution of β^ts2sls is given by

n1(β^ts2sls−β)⟶dN(0,Vβ);

(10) $$V_{\beta }=C\left(V_{\pi_{y1}}+\alpha \left(\beta^{\prime }⊗I_{k_{z}}\right)V_{\pi_{x2}}\left(\beta ⊗I_{k_{z}}\right)\right)C^{\prime }=CV_{\pi_{y1}}C^{\prime }+\alpha \left(\beta^{\prime }⊗C\right)V_{\pi_{x2}}\left(\beta ⊗C^{\prime }\right)\text{,}$$

where

(11) $$C={\left(\Pi_{x2}^{\prime }Q_{zz1}\Pi_{x2}\right)}^{-1}\Pi_{x2}^{\prime }Q_{zz1}\text{.}$$

We can obtain an estimator for the asymptotic variance of β^ts2sls as follows. Let Va^r(π^y1) and Va^r(π^x2) be estimators of the asymptotic variances of π^y1 and π^x2, in the sense that plim(n1Va^r(π^y1))=Vπy1 and plim(n2Va^r(π^x2))=Vπx2. Let C^ be the matrix of least squares coefficients from the regressions of the columns of $Z_{1}$ on X^1. As plim(C^)=plim((X^1′X^1)−1X^1′Z1)=C, an estimator of the asymptotic variance of β^ts2sls is given by

(12) Va^r(β^ts2sls)=C^Va^r(π^y1)C^′+(β^ts2sls′⊗C^)Va^r(π^x2)(β^ts2sls⊗C^′),

as

n1Va^r(β^ts2sls)=C^(n1Va^r(π^y1))C^′+n1n2(β^ts2sls′⊗C^)(n2Va^r(π^x2))(β^ts2sls⊗C^′)⟶pVβ.

When the model is just identified, $k_{z}=k_{x}$, then C^=Π^x2−1. When furthermore $k_{x}=k_{z}=1$, (12) reduces to the simple expression

Va^r(β^ts2sls)=(Va^r(π^y1)+β^ts2sls2Va^r(π^x2))/π^x22,

with β^ts2sls=π^y1π^x2, which is identical to the expression obtained using the delta method as in Dee and Evans (2003).

Specifying Va^r(π^y1) and Va^r(π^x2) in (12) as being robust to general forms of heteroskedasticity results in a robust variance estimator for β^ts2sls. A small Monte Carlo exercise reported in the Appendix confirms that our asymptotic results reflect the behaviour of the TS2SLS estimator. Although we have here an i.i.d. cross-sectional setup, the results generalise to e.g. cluster-robust variances straightforwardly.

## 4 GMM

Assuming conditional homoskedasticity for both $u_{1i}$ and $v_{2j}$ such that

$$E\left(u_{1i}^{2}|z_{1i}\right)=\sigma_{u}^{2}\;\text{and}\;E\left(v_{2j}v_{2j}^{\prime }|z_{2j}\right)=\Sigma_{v}\text{,}$$

we have that

$$V_{\pi_{y1}}=\sigma_{u}^{2}Q_{zz1}\;\text{and}\;V_{\pi_{x2}}=\Sigma_{v}⊗Q_{zz2}^{-1}\text{,}$$

and hence

$$V_{\beta }=\sigma_{u}^{2}{\left(\Pi_{x2}^{\prime }Q_{zz1}\Pi_{x2}\right)}^{-1}+\alpha \beta^{\prime }\Sigma_{v}\beta CQ_{zz2}^{-1}C^{\prime }\text{.}$$

The variance estimator (12) is then

(13) Va^r(β^ts2sls)=σ^u2(X^1′X^1)−1+β^ts2sls′Σ^vβ^ts2slsC^(Z2′Z2)−1C^′,

with σ^u2=(y1−Z1π^y1)′(y1−Z1π^y1)/n1 and Σ^v=(X2−Z2Π^x2)′(X2−Z2Π^x2)/n2.

Inoue and Solon (2010) derive $V_{\beta }$ from the limiting distribution of the optimal GMM estimator using moment conditions

(14) $$E\left[z_{1i}\left(y_{1i}-z_{1i}^{\prime }\Pi_{x2}\beta \right)\right]=0\text{;}$$

(15) $$E\left[z_{2j}⊗\left(x_{2j}-\Pi_{x2}^{\prime }z_{2j}\right)\right]=0\text{,}$$

and weight matrix

[Va^r(π^y1)00Va^r(π^x2)]=[σ^u2(Z1′Z1)−100Σ^v⊗(Z2′Z2)−1].

Let $\psi ={\left(\begin{array}{cc} \beta^{\prime } & \pi_{x2}^{\prime } \end{array}\right)}^{\prime }$, thenthis GMM estimator is the same as the minimum distance estimator

ψ˜=argminβ,πx2(π^y1−Πx2βπ^x2−πx2)′[(Va^r(π^y1))−100(Va^r(π^x2))−1](π^y1−Πx2βπ^x2−πx2).

Unless the model is just identified, β˜≠β^ts2sls, but their limiting distributions are the same. This is a situation similar to that of the LIML and 2SLS estimators in the standard IV model. When the model is overidentified, the TS2SLS estimator itself cannot be obtained as a GMM estimator. The limiting variance of $\sqrt{n_{1}}\left(\overset{˜}{\beta }-\beta \right)$ is obtained from the limiting variance of $\sqrt{n_{1}}\left(\overset{˜}{\psi }-\psi \right)$. Inoue and Solon (2010) imposed $Q_{zz1}=Q_{zz2}$ and obtained the variance as

$$V_{\beta ,IS}=\left(\sigma_{u}^{2}+\alpha \beta^{\prime }\Sigma_{v}\beta \right){\left(\Pi_{x2}^{\prime }Q_{zz1}\Pi_{x2}\right)}^{-1}$$

and their variance estimator is given by

Va^rIS(β^ts2sls)=(σ˜u2+n1n2β^ts2sls′Σ^vβ^ts2sls)(X^1′X^1)−1,

where σ˜u2=(y1−X^1β^ts2sls)′(y1−X^1β^ts2sls)/n1. Apart from this difference in the estimation of $\sigma_{u}^{2}$, the main difference is the imposition that $Q_{zz1}=Q_{zz2}$. Although this is justified asymptotically given the Assumptions A1–A3, the finite sample variance of π^x2 in (12) is clearly more naturally estimated by Σ^v⊗(Z2′Z2)−1 than by Σ^v⊗(n2n1Z1′Z1)−1. Also, for the example in footnotes 3 and 2 in Inoue and Solon, 2010, Inoue and Solon, 2005 respectively, when $E\left(z_{1i}x_{1i}^{\prime }\right)=cE{\left(z_{2j}x_{2j}\right)}^{\prime }$ and $E\left(z_{1i}z_{1i}^{\prime }\right)=cE{\left(z_{2j}z_{2j}\right)}^{\prime }$, with c≠1, then the TS2SLS estimator is consistent and asymptotically normally distributed but n1Va^rIS(β^ts2sls) is no longer a consistent estimator of the variance of the limiting distribution, whereas n1Va^r(β^ts2sls) is.

Inoue and Solon (2010) did not derive the robust variance of $\overset{˜}{\beta }$. Although this can be obtained from the robust variance of $\overset{˜}{\psi }$, the matrix expressions involved are quite cumbersome. Arellano and Meghir (1992) similarly considered the robust variance of the GMM estimator $\overset{˜}{\psi }$ but also did not derive a variance estimator for $\overset{˜}{\beta }$ separately. One can of course simply obtain robust standard errors for $\overset{˜}{\psi }$ and hence $\overset{˜}{\beta }$ using GMM routines that can estimate the parameters using the nonlinear and linear moment conditions (14), (15). These estimates are then obtained using iterative methods, and for just-identified models this produces the TS2SLS estimator with robust standard errors. For overidentified models, the efficient two-step GMM estimator for ψ can then also be obtained together with a Hansen test for the validity of the moment conditions. We present Stata code for this GMM estimation procedure in the Appendix (see Appendix A).

## 5 Generalising the result

Although we derived the results in Section  3 for the standard TS2SLS estimator, the limiting distribution results (17) and (18) in the Appendix (see Appendix A) apply more generally. Indeed, the only aspect in $V_{\theta }$ that is particular to this specific two-sample setup is the zero covariance between π^y1 and π^x2, due to the samples being independent.

Consider as a generalisation a model with three explanatory variables $x_{1}$, $x_{2}$ and $x_{3}$. Using the same notational convention as before, in sample 1 we observe ${\left\{y_{1i},x_{11i},x_{31i},z_{1i}^{\prime }\right\}}_{i=1}^{n_{1}}$. In sample 2 we observe ${\left\{x_{22j},x_{32j},z_{2j}^{\prime }\right\}}_{j=1}^{n_{2}}$. In this case, $x_{1}$ is only observed in sample 1, $x_{2}$ is only observed in sample 2, whereas $x_{3}$ is observed in both samples. Let $Z={\left(\begin{array}{cc} Z_{1}^{\prime } & Z_{2}^{\prime } \end{array}\right)}^{\prime }$and $x_{3}={\left(\begin{array}{cc} x_{31}^{\prime } & x_{32}^{\prime } \end{array}\right)}^{\prime }$, then the reduced form and first-stage OLS estimators are given by

π^y1=(Z1′Z1)−1Z1′y1;π^x11=(Z1′Z1)−1Z1′x11

π^x22=(Z2′Z2)−1Z2′x22;π^x3=(Z′Z)−1Z′x3.

Let Π^x=[π^x11π^x22π^3], then the two-sample IV estimator is given by

β^2s=(Π^x′Z1′Z1Π^x)−1Π^x′Z1′Z1π^y1.

We differentiate this estimator from the standard two-sample setup above and reserve the name β^ts2sls for that particular setup. Under Assumptions A1–A7, the limiting distribution is as in (17), but as θ^=(π^y1′vec(Π^x)′), the variance $V_{\theta }$ differs from the standard setup as there is a different covariance structure. There are non-zero covariances between π^y1 and π^x11; π^x11 and π^x3; and π^x11 and π^x3, whereas the covariances between π^y1 and π^x22; and π^x11 and π^x22 are zero. From (18), an estimator for the asymptotic variance is given by

(16) Va^r(β^2s)=(δ^′⊗C^)Va^r(θ^)(δ^⊗C^′),

where δ^=(1−β^2s′)′ and C^=(X^1′X^1)−1X^1′Z1=(Π^x′Z1′Z1Π^x)−1Π^x′Z1′Z1.

For the standard TS2SLS setup and the more general structures, one can obtain the robust variance estimates using standard routines. We give Stata code for two examples in the Appendix (see Appendix A). The structure of the algorithm for the general case is:

1. Estimate the reduced form and first-stage parameters by OLS, obtain the predicted values X^1 and a robust variance estimate for θ^=(π^y1′π^x2′)′, the matrix Va^r(θ^). In Stata, the latter can be obtained using the ‘gmm’ or the ‘suest’ routine.
2. Regress $y_{1}$ on X^1 to obtain the TS2SLS estimator.
3. Regress the columns of $Z_{1}$ on X^1 and collect the parameter estimates in the matrix C^.
4. Calculate Va^r(β^2s) by the matrix expression in (16).
5. Some adjustments have to be made when parameters on exogenous variables and the constant are included in the estimation. These are detailed in the code in the Appendix (see Appendix A).

## Appendix A Supplementary material

MMC S1 Appendix A. Supplementary material.

## Footnotes

Appendix A Supplementary material related to this article can be found online at http://dx.doi.org/10.1016/j.econlet.2016.06.033.
