# Supplementary material for: Robust inference for the Two-Sample 2SLS estimator
Source: Econ Lett. 2016 Sep;146:50–4. doi: 10.1016/j.econlet.2016.06.033 (PMC5026329; doi:10.1016/j.econlet.2016.06.033)
Supplement: MMC S1 — Appendix A. Supplementary material. [file mmc1.pdf]

A Appendix

A.1 Derivation of Limiting Distribution

The limiting distribution of $\widehat{\beta}_{ts2sls}$ can be obtained as follows, alternative methods like the delta method or Newey (1984) give identical results. Rewrite the reduced form model for y_{1i} as

$$\begin{aligned} y_{1i} &= z_{1i}'\pi_{y1} + u_{1i} = z_{1i}'\Pi_{x1}\beta + u_{1i} \\ &= z_{1i}'\widehat{\Pi}_{x2}\beta + u_{1i} - z_{1i}'\left(\widehat{\Pi}_{x2} - \Pi_{x1}\right)\beta \\ &= \widehat{x}_{1i}'\beta + u_{1i} - z_{1i}'\left(\widehat{\Pi}_{x2} - \Pi_{x2}\right)\beta, \end{aligned}$$

where the last equality holds as $\Pi_{x2} = \Pi_{x1}$ by the assumptions. Then

$$\begin{aligned} \widehat{\beta}_{ts2sls} &= \left(\widehat{X}_1'\widehat{X}_1\right)^{-1}\widehat{X}_1 y_1 \\ &= \beta + \left(\widehat{X}_1'\widehat{X}_1\right)^{-1}\widehat{X}_1'\left(u_1 - Z_1\left(\widehat{\Pi}_{x2} - \Pi_{x2}\right)\beta\right), \end{aligned}$$

where u_1 is the n_1 vector (u_{1i}). For the limiting distribution,

$$\sqrt{n_1}\left(\widehat{\beta}_{ts2sls} - \beta\right) = \left(\frac{1}{n_1}\widehat{X}_1'\widehat{X}_1\right)^{-1}\frac{1}{\sqrt{n_1}}\widehat{X}_1'\left(u_1 - Z_1\left(\widehat{\Pi}_{x2} - \Pi_{x2}\right)\beta\right).$$

As

$$\begin{aligned} &\frac{1}{\sqrt{n_1}}\widehat{X}_1'\left(u_1 - Z_1\left(\widehat{\Pi}_{x2} - \Pi_{x2}\right)\beta\right) \\ &= \frac{1}{\sqrt{n_1}}\widehat{\Pi}_{x2}'Z_1'\left(u_1 - Z_1\left(\widehat{\Pi}_{x2} - \Pi_{x2}\right)\beta\right) \\ &= \frac{1}{\sqrt{n_1}}\widehat{\Pi}_{x2}'Z_1'Z_1\left(\left(Z_1'Z_1\right)^{-1}Z_1'u_1 - \left(\widehat{\Pi}_{x2} - \Pi_{x2}\right)\beta\right) \\ &= \widehat{\Pi}_{x2}'\left(\frac{1}{n_1}Z_1'Z_1\right)\left(\sqrt{n_1}\left(\widehat{\pi}_{y1} - \pi_{y1}\right) - \sqrt{n_1}\left(\widehat{\Pi}_{x2} - \Pi_{x2}\right)\beta\right), \end{aligned}$$

and

$$\begin{aligned} \left(\widehat{\Pi}_{x2} - \Pi_{x2}\right)\beta &= \operatorname{vec}\left(\left(\widehat{\Pi}_{x2} - \Pi_{x2}\right)\beta\right) \\ &= \left(\beta' \otimes I_{k_z}\right)\operatorname{vec}\left(\widehat{\Pi}_{x2} - \Pi_{x2}\right) \\ &= \left(\beta' \otimes I_{k_z}\right)\left(\widehat{\pi}_{x2} - \pi_{x2}\right), \end{aligned}$$

it follows that

$$\sqrt{n_1}\left(\widehat{\beta}_{ts2sls}-\beta\right)=\left(\frac{1}{n_1}\widehat{X}_1'\widehat{X}_1\right)^{-1}\widehat{\Pi}_{x2}'\left(\frac{1}{n_1}Z_1'Z_1\right)\left(\delta'\otimes I_{k_z}\right)\sqrt{n_1}\left(\widehat{\theta}-\theta\right),$$

where $\delta=\left(\begin{array}{cc}1 & -\beta'\end{array}\right)'$.

As

$$\text{plim}\left(\frac{1}{n_1}\widehat{X}_1'\widehat{X}_1\right)^{-1}\left(\widehat{\Pi}_{x2}'\left(\frac{1}{n_1}Z_1'Z_1\right)\right)=\left(\Pi_{x2}'Q_{zz1}\Pi_{x2}\right)^{-1}\Pi_{x2}'Q_{zz1}=C,$$

it follows that

$$\sqrt{n_1}\left(\widehat{\beta}_{ts2sls}-\beta\right)\overset{d}{\longrightarrow}N\left(0,V_\beta\right), \tag{17}$$

where

$$\begin{aligned}V_\beta &= C\left(\delta'\otimes I_{k_z}\right)V_\theta\left(\delta\otimes I_{k_z}\right)C' \\ &= \left(\delta'\otimes C\right)V_\theta\left(\delta\otimes C'\right) \\ &= C\left(V_{\pi_{y1}}+\alpha\left(\beta'\otimes I_{k_z}\right)V_{\pi_{x2}}\left(\beta\otimes I_{k_z}\right)\right)C' \\ &= CV_{\pi_{y1}}C'+\alpha\left(\beta'\otimes C\right)V_{\pi_{x2}}\left(\beta\otimes C'\right).\end{aligned} \tag{18}$$

A.2 Some Monte Carlo Results

We generate data according to the standard setup for the TS2SLS estimator above. The parameters in model (19) are set to $\beta_1=0.3$, $\beta_2=-0.1$, $\beta_w=0.1$ and $\beta_0=0.2$. Further, $w_i\sim N(0,1)$, and

$$\begin{aligned}x_1 &= Z\pi_1+w\pi_{w1}+\pi_{01}+v_1 \\ x_2 &= Z\pi_2+w\pi_{w2}+\pi_{02}+v_2\end{aligned}$$

where $Z=\left[\begin{array}{ccc}z_1 & z_2 & z_3\end{array}\right]$ and $z_i\sim N(0,I_3)$. The parameters for x_1 are given by $(0.4,0.6,-0.2,0.4,0.2)$, those for x_2 by $(0.2,-0.2,0.6,0.4,-0.6)$. We draw

$$u_i=\left(\begin{array}{c}u_{1i}\\ u_{2i}\\ u_{3i}\end{array}\right)\sim N\left(\left(\begin{array}{c}0\\0\\0\end{array}\right),\left(\begin{array}{ccc}1 & \rho_1 & \rho_2\\ \rho_1 & 1 & \rho_1\rho_2\\ \rho_2 & \rho_1\rho_2 & 1\end{array}\right)\right)$$

with $\rho_1=0.3$ and $\rho_2=-0.2.$, and set $\varepsilon_i=u_{1i}\sqrt{\exp\left(\gamma_\varepsilon z_1\right)}$, $v_{1i}=u_{2i}\sqrt{\exp\left(z_i'\gamma\right)}$ and $v_{2i}=u_{3i}\sqrt{\exp\left(z_i'\gamma\right)}$.

The first design is homoskedastic and hence $\gamma_\varepsilon = 0$ and $\gamma = 0$. The second design is heteroskedastic, with $\gamma_\varepsilon = 1.5$, and $\gamma = (0.5, 0.8, -0.3)'$. We standardise the n-vectors ε, v_1 and v_2 such the square of their L_2-norms are equal to n, where $n = n_1 + n_2$. Estimation results for these two designs are presented in Table 1. Sample sizes are $n_1 = 500$ and $n_2 = 1000$ for both designs. Results are presented from 10,000 MC replications. The table reports the means and standard deviations of the TS2SLS estimates for β_1 and β_2, the means of the non-robust and robust standard errors, plus the rejection frequencies of the Wald tests, testing $H_0 : \beta_1 = 0.3$, and $H_0 : \beta_2 = -0.1$ respectively, at the 5% nominal size. The results clearly show that the means of the robust standard errors are very close to the standard deviation of the TS2SLS estimates for both designs, whereas the non-robust standard errors underestimate the variability of the estimates in the heteroskedastic design. This is reflected in the behaviour of the Wald tests. Those based on the robust variance estimates have correct size, whereas those based on the non-robust variance estimates overreject the null in the heteroskedastic design.

Table 1. Monte Carlo results for the TS2SLS estimator

Design	mean	std dev	mean se	mean rob se	Wald	rob Wald
Homoskedastic						
β_1	0.300	0.075	0.074	0.074	0.049	0.051
β_2	-0.099	0.086	0.083	0.083	0.054	0.055
Heteroskedastic						
β_1	0.301	0.102	0.072	0.099	0.155	0.052
β_2	-0.099	0.099	0.082	0.096	0.102	0.054

Notes: results from 10,000 MC replications. Rej. freq. of Wald tests at 5% nominal size. Sample sizes n_1= 500, n_2= 1000

A.3 Stata Code

A.3.1 TS2SLS

In the first example, we want to estimate the following model

$$y = x_1\beta_1 + x_2\beta_2 + w\beta_w + \beta_0 + \varepsilon. \tag{19}$$

We are in the standard setup for the TS2SLS estimator. In sample 1, we have observations on the variables y, w, z_1, z_2 and z_3. In sample 2, we have observations on the variables x_1, x_2, w, z_1, z_2 and z_3. Simple Stata syntax to compute the TS2SLS estimator and the non-robust and robust standard errors is given below.

```
use sample2.dta, clear
gen const = 1

qui gmm (x1 - {xb1: z1 z2 z3 w const}) ///
     (x2 - {xb2:z1 z2 z3 w const}), ///
     instruments(1 2: z1 z2 z3 w) ///
     winit(unadjusted,independent) onestep  ///
     deriv(1/xb1 = -1) ///
     deriv(2/xb2 = -1)
mat Vx2het = e(V)      /*Robust variance estimate of pix2*/

qui sureg (x1 x2 = z1 z2 z3 w )
mat Vx2hom = e(V)      /*Non-robust variance estimate of pix2*/

use sample1.dta, clear

/*Generating predicted X*/
qui predict x1h, equation(x1)
qui predict x2h, equation(x2)

scalar kx = 2       /*Number of predicted variables, here x1 and x2*/
scalar ke = 2       /*Number of exogenous variables, here w and constant*/

qui reg y z1 z2 z3 w
mat Vy1hom = e(V)*e(df_r)/_N   /*Non-robust variance estimate of piy1,*/
                               /*without degrees of freedom correction*/
qui reg y z1 z2 z3 w, rob
mat Vy1het = e(V)*e(df_r)/_N   /*Robust variance estimate of piy1,*/
                               /*without degrees of freedom correction*/

/*TS2SLS estimator*/
qui reg y x1h x2h w
mat b2s = e(b)
mat b2sx = b2s[1,1..kx]'         /*Selecting beta for predicted X only*/

/*Constructing C hat*/
qui reg z1 x1h x2h w
mat ch = e(b)'
qui reg z2 x1h x2h w
mat ch = ch,e(b)'
qui reg z3 x1h x2h w
mat ch = ch,e(b)'
mat ch = ch,(J(kx,ke,0)\I(ke))        /*Adjusting ch for the exogenous variables*/


/*Calculating non-robust standard errors*/
mat var1hom =  ch*Vy1hom*ch' + (b2sx' # ch)*Vx2hom*(b2sx # ch')
mat seb2shom = vecdiag(cholesky(diag(vecdiag(var1hom))))'

/*Calculating robust standard errors*/
mat var1het =  ch*Vy1het*ch' + (b2sx' # ch)*Vx2het*(b2sx # ch')
```

```
mat seb2shet = vecdiag(cholesky(diag(vecdiag(var1het))))'

/*Displaying the results*/
mat res = b2s',seb2shom,seb2shet
mat colnames res = b_ts2sls se "rob se"
mat rownames res = x1 x2 w _cons
matlist res
```

A.3.2 A Generalisation

In the second example, we are interested in estimating the model

$$y = x_1\beta_1 + x_2\beta_2 + x_3\beta_3 + w\beta_w + \beta_0 + \varepsilon.$$

We now observe in sample 1 the variables y, x_1, x_3, w, z_1, z_2, z_3 and z_4. In sample 2 we observe the variables x_2, x_3, w, z_1, z_2, z_3 and z_4. The Stata code for computing the Two-Sample estimator and the robust standard errors is as follows.

```
/*Merging Sample1 and Sample2 data on identifier id*/
use sample1.dta, clear
qui merge 1:1 id using sample2.dta

/*Generating Var(theta) and predicted X*/
qui reg y z1 z2 z3 z4 w
est store eqn_y
qui reg x1 z1 z2 z3 z4 w
est store eqn_x1
qui predict x1h
qui reg x2 z1 z2 z3 z4 w
est store eqn_x2
qui predict x2h
qui reg x3 z1 z2 z3 z4 w
est store eqn_x3
qui predict x3h

qui suest eqn_y eqn_x1 eqn_x2 eqn_x3

mat var = e(V)*(_N-1)/_N     /*Robust variance estimate of theta*/
                             /*without degrees of freedom correction*/

/*Selecting rows and columns from var associated with theta*/
mata
kz = 6       /*Total number of instruments, here z1, z2, z3, z4, w and const*/
kyx = 4      /*Number of variables in X, here x1, x2 and x3, plus 1 for y*/
sel = range(1,kz,1)
j = 2
while (j<=kyx)
{
```

```
ss = range((j-1)*kz+j,j*kz+(j-1),1)
sel = sel\ss
j = j+1
}

var = st_matrix("var")
var = var[sel,sel]
st_matrix("Vthetahet",var)
end

/*Selecting Sample1 data and variables*/
/*to compute TS estimator and variance*/
drop if y==.
keep y x1h x2h x3h w z1 z2 z3 z4

scalar kx = 3       /*Number of predicted variables, here x1, x2 and x3*/
scalar ke = 2       /*Number of exogenous variables, here w and constant*/

/*TS Estimator*/
qui reg y x1h x2h x3h w
mat b2s = e(b)
mat b2sx = b2s[1,1..kx]'           /*Selecting beta for predicted X only*/

/*Constructing C hat*/
qui reg z1 x1h x2h x3h w
mat ch = e(b)'
qui reg z2 x1h x2h x3h w
mat ch = ch,e(b)'
qui reg z3 x1h x2h x3h w
mat ch = ch,e(b)'
qui reg z4 x1h x2h x3h w
mat ch = ch,e(b)'
mat ch = ch,(J(kx,ke,0)\I(ke))        /*Adjusting ch for the exogenous variables*/

/*Calculating robust standard errors*/
mat delta = 1\-b2sx
mat var1het = (delta' # ch)*Vthetahet*(delta # ch')
mat seb2shet = vecdiag(cholesky(diag(vecdiag(var1het))))'

/*Displaying the results*/
local names = "x1 x2 x3 w _cons"
mat colnames b2s = `names'
mat colnames var1het = `names'
mat colnames var1het = _:
mat rownames var1het = `names'
mat rownames var1het = _:

cap prog drop output2s
prog output2s, eclass
eret post b2s var1het
eret local depvar y
eret local vcetype Robust
```

```
eret dis
end

output2s
```

A.3.3 GMM

Stata code for the nonlinear GMM estimator for the example as in Section A.3.1.

```
use sample2.dta, clear
qui sureg (x1 x2 = z1 z2 z3 w)
mat Vpx2 = e(V)

use sample1.dta, clear
qui reg y z1 z2 z3 w
matrix Vpy1 = e(V)

/*vyvxmat is weightmatrix for optimal GMM estimator under conditional homoskedasticity*/
mat vyvxmat = Vpy1,J(rowsof(Vpy1),colsof(Vpx2),0)
mat vyvxmat = vyvxmat\(J(rowsof(Vpx2),colsof(Vpy1),0),Vpx2)

/*s1 is sample 1 identifier*/
gen s1 = 1

/*Merging Sample1 and Sample2 data on identifier id*/
/*No overlap in id here*/
qui merge 1:1 id using sample2.dta

/*s2 is sample 2 identifier*/
replace s1 = 0 if s1==.
gen s2 = 1-s1

/*setting values of y and x to zero in samples where missing*/
replace y = 0 if s2==1
replace x1 = 0 if s1==1
replace x2 = 0 if s1==1

/*instruments with different names for different samples, with zero values elsewhere*/
gen z11 = z1*s1
gen z12 = z1*s2
gen z21 = z2*s1
gen z22 = z2*s2
gen z31 = z3*s1
gen z32 = z3*s2
gen w1 = w*s1
gen w2 = w*s2

#delimit ;
gmm (y - {b1}*({p11}*z11+{p12}*z21+{p13}*z31+{p1w}*w1+{p10}*s1)
   - {b2}*({p21}*z11+{p22}*z21+{p23}*z31+{p2w}*w1+{p20}*s1)
   - {bw}*w1-{b0}*s1)
```

```
(x1 - {p11}*z12-{p12}*z22-{p13}*z32-{p1w}*w2-{p10}*s2)
(x2 - {p21}*z12-{p22}*z22-{p23}*z32-{p2w}*w2-{p20}*s2),
 instruments(1:z11 z21 z31 w1 s1, nocons)
 instruments(2 3:z12 z22 z32 w2 s2, nocons)
 winit(vyvxmat) onestep
 deriv(1/b1 = -({p10}*s1+{p1w}*w1+{p11}*z11+{p12}*z21+{p13}*z31))
 deriv(1/b2 = -({p20}*s1+{p2w}*w1+{p21}*z11+{p22}*z21+{p23}*z31))
 deriv(1/bw = -w1)
 deriv(1/b0 = -s1)
 deriv(1/p11 = -{b1}*z11)
 deriv(1/p12 = -{b1}*z21)
 deriv(1/p13 = -{b1}*z31)
 deriv(1/p1w = -{b1}*w1)
 deriv(1/p10 = -{b1}*s1)
 deriv(1/p21 = -{b2}*z11)
 deriv(1/p22 = -{b2}*z21)
 deriv(1/p23 = -{b2}*z31)
 deriv(1/p2w = -{b2}*w1)
 deriv(1/p20 = -{b2}*s1)
 deriv(2/p11 = -z12)
 deriv(2/p12 = -z22)
 deriv(2/p13 = -z32)
 deriv(2/p1w = -w2)
 deriv(2/p10 = -s2)
 deriv(3/p21 = -z12)
 deriv(3/p22 = -z22)
 deriv(3/p23 = -z32)
 deriv(3/p2w = -w2)
 deriv(3/p20 = -s2);

/*use twostep option and ‘estat overid’ command for efficient GMM and Hansen test*/
```

References

Newey, W.K. (1984), A method of moments interpretation of sequential estimators, *Economics Letters* 14, 201-206.
